# Supplementary material for: Ageing Curtails the Diversity and Functionality of Nascent CD8+ T Cell Responses against SARS-CoV-2
Source: Vaccines (Basel). 2023 Jan 11;11(1):154. doi: 10.3390/vaccines11010154 (PMC9867380; doi:10.3390/vaccines11010154)
Supplement: Supplementary file 1 [file vaccines-11-00154-s001.zip › vaccines-2100417-supplementary.pdf]

vaccines

MDPI

Supplementary Materials:

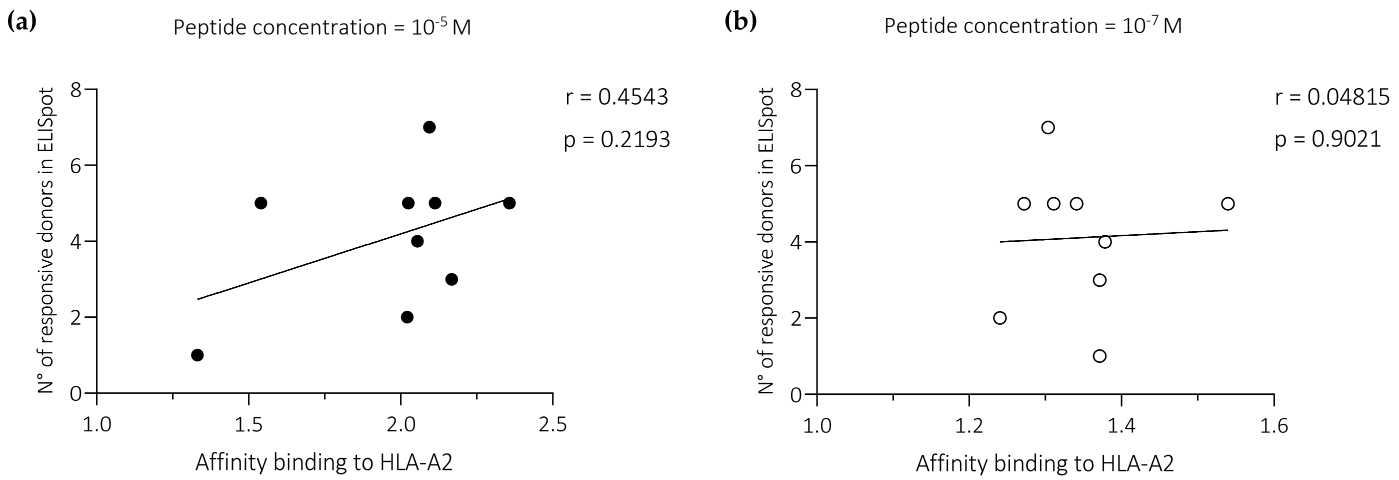

Figure S1. Correlation between peptide binding affinity and immunoprevalence. Affinity binding to HLA-A2 was calculated for each peptide at 10^{-5} M (**a**) or 10^{-7} M (**b**) and correlated with the number of responsive donors as determined using ELISpot assays to capture IFN-γ. Significance was determined using the Pearson correlation coefficient.
